# circ-Sirt1 Decelerates Senescence by Inhibiting p53 Activation in Vascular Smooth Muscle Cells, Ameliorating Neointima Formation

**DOI:** 10.3389/fcvm.2021.724592

**Published:** 2021-12-17

**Authors:** Peng Kong, Chang-Lin Li, Yong-Qing Dou, Li Cao, Xiao-Yun Zhang, Wen-Di Zhang, Ze-Qi Bi, Zu-Yi Peng, An-Qi Yan, Mei Han

**Affiliations:** ^1^Key Laboratory of Medical Biotechnology of Hebei Province, Key Laboratory of Neural and Vascular Biology of Ministry of Education, Department of Biochemistry and Molecular Biology, College of Basic Medicine, Hebei Medical University, Shijiazhuang, China; ^2^College of Integrative Medicine, Hebei University of Chinese Medicine, Shijiazhuang, China

**Keywords:** circ-Sirt1, senescence, vascular smooth muscle cells, neointimal hyperplasia, p53

## Abstract

Vascular smooth muscle cell (VSMC) senescence is a major driver of neointimal formation. We have demonstrated that circ-Sirt1 derived from the SIRT1 gene suppressed VSMC inflammation and neointimal formation. However, the effect of circ-Sirt1 inhibiting inflammation on VSMC senescence during neointimal hyperplasia remains to be elucidated. Here, we showed that circ-Sirt1 was highly expressed in young and healthy arteries, which was decreased in aged arteries and neointima of humans and mice. Overexpression of circ-Sirt1 delayed Ang II-induced VSMC senescence *in vitro* and ameliorated neointimal hyperplasia *in vivo*. Mechanically, circ-Sirt1 inhibited p53 activity at the levels of transcription and post-translation modulation. In detail, circ-Sirt1, on the one hand, interacted with and held p53 to block its nuclear translocation, and on the other hand, promoted SIRT1-mediated p53 deacetylation and inactivation. In conclusion, our data suggest that circ-Sirt1 is a novel p53 repressor in response senescence-inducing stimuli, and targeting circ-Sirt1 may be a promising approach to ameliorating aging-related vascular disease.

## Introduction

Aging is a biological process caused by the accumulation of senescent cells that are defined as an irreversible loss of proliferation potential (1). Vascular smooth muscle cells (VSMCs), as the major components of the blood vessel wall, play crucial roles in both physiological and pathological processes such as restenosis and atherosclerosis (2). It has been known that VSMC senescence promotes neointimal formation *via* increase in migration, oxidative stress, inflammation and collagen deposition in the intima in response to vascular injury (3, 4). Although several studies have shown evidence for the contribution of senescent cells to the development of vascular proliferative diseases, the mechanism by which aging exaggerates neointima formation after vascular injury has, so far, remained elusive.

Circular RNAs, a novel class of endogenous non-coding RNAs, are characterized by their covalently closed loop structures without a 5' cap or a 3' poly A tail. Recently, a global accumulation of circRNAs during aging has been identified across different species, indicating a potential role as a causal factor in aging and age-related disease (5–7). Our previous study demonstrated that circ-Sirt1 derived from the SIRT1 gene suppressed vascular inflammation and neointimal formation by direct interaction with NF-κB p65 and promoting expression of SIRT1, an NAD^+^-dependent deacetylase, through competitive binding to miR-132/212 (8). It has been known that inflammatory response can induce dedifferentiation VSMCs from a contractile phenotype to a proliferative and synthetic state. Replicative senescence is highly relevant in the context of VSMC proliferation because the generation of all the VSMC-derived cells in advanced plaques by proliferative expansion would probably cause replicative senescence (9). For example, platelet derived growth factor (PDGF) as a mitogenic growth factor promotes the proliferation of cells while also induces senescence (10), and aging increases the ability of VSMCs to proliferate and decreases susceptibility to apoptosis (11). SIRT1 is considered to be an anti-aging molecule involved in the regulation of senescence-associated calcification, atherosclerosis, fibrous cap stability and medial degeneration (12–14). Targets for SIRT1 deacetylation are key components of the cellular senescence. However, the effect of circ-Sirt1 inhibiting vascular inflammation on cellular senescence during neointimal hyperplasia remains to be elucidated.

In this study, we demonstrated that the level of circ-Sirt1 was decreased in the arteries of aged mice and the neointima. Overexpression of circ-sirt1 suppressed VSMC senescence, accompanied with reduced neointimal formation. Our findings suggest that targeting circ-Sirt1 may be a promising approach to ameliorating VSMC senescence.

## Materials and Methods

### The Carotid Artery Ligation Injury and Adenoviral Infection

The C57BL/6J mice (2- to 3-month-old male mice as young mice and 18- to 24-month-old male mice as aged mice) were purchased from Jackson Laboratory (Bar Harbor, ME). The carotid artery injury was induced by complete ligation of the left common carotid artery as previously described (15). A sham operation was performed on the contralateral right common carotid artery. For infection, after left carotid artery ligation, adenoviral vector-containing solution (1 × 10^10^ pfu/mL) was suspended together in 50 μL pluronic-F127 gel (Sigma-Aldrich; 25% wt/vol) and applied around the carotid artery. Carotid arteries were harvested at 14 days after ligation.

This study was performed *via* a protocol approved by the Institutional Animal Care and Use Committee of Hebei Medical University, in accordance with the Guide for the Care and Use of Laboratory Animals.

### Human Arterial Tissues

Human vascular samples were obtained from 15 patients undergoing nephrectomy at the Fourth Hospital of Hebei Medical University (Shijiazhuang, China). Among them, there were 5 patients with atherosclerosis and 5 control samples from normal patients, as well as 5 patients who had hypertension at least 10 years and managed blood pressure by using hypotensor.

The Ethical Committee of Hebei Medical University approved all protocols using human samples. All patients or their relatives provided written informed consent prior to their participation in the study (2020032).

### Hematoxylin and Eosin (H&E) Staining

Carotid arteries were harvested at 14 days after ligation. The animals were euthanized by an intraperitoneal injection of ketamine (80 mg/kg)/xylazine (5 mg/kg). The left ventricle was cannulated and perfused with phosphate buffered saline (PBS) containing heparin, and then perfused and fixed with 4% paraformaldehyde in PBS under physiological pressure. The left carotid artery was then removed, further fixed for 12 h, and optimum cutting temperature (OCT)-embedded without further dissection. Serial sections (5 μm thick) were obtained at 500 μm proximal to the ligation site. The cross-sectional areas of the intima and media were measured in H&E-stained sections in a blinded manner by a single observer using Image Pro Plus 6.0 software (Media Cybernetics). A mean value was determined from at least 3 sections for each animal. Neointima formation was determined as the ratio of the intimal area to the medial area (I/M).

### SA-β-Gal Staining

SA-β-gal activity was detected using the Senescent Cells Staining Kit (Cell Signaling Technology) as previously described (16). The SA-β-gal signals were analyzed using Image J software.

### Cell Culture, Treatment, and Migration Assays

Thoracic aortas were dissected from mice and incubated in low glucose Dulbecco's-modified Eagle's medium (DMEM, Invitrogen) containing 1 mg/mL collagenase II (Sigma) for 1 h at 37°C. After removal of the adventitia, the aortas were minced and incubated in DMEM containing 2 mg/mL collagenase II and 0.5 mg/mL elastase (Sigma) for 1 h at 37°C. After centrifugation to remove collagenase digestion solution, the isolated cells were resuspended in DMED supplemented with 10% fetal bovine serum (FBS; Gibco), 100 U/ml penicillin and 100 μg/ml streptomycin. Passage 3 to 8 cells were used in the experiments. The VSMCs were maintained at 37°C in a humidified atmosphere containing 5% CO_2_, and cells at 40–60% confluence were used in the experiments. Before stimulation with Ang II (100 nM, Sigma) or PDGF-BB (20 ng/mL, R&D Systems) the VSMCs were incubated in serum free medium for 24 h. To inhibit the activity of SIRT1, cells were pretreatment with the inhibitor Ex527 (20 μmol/L; Cayman).

The migration of VSMCs was evaluated by performing a cell-wounding assay. Cells grown to 100% confluence on glass slides were scraped off the slides with a cell scraper to create a 3-mm-wide wound, overexpressed or knockdown circ-Sirt1 following treated with Ang II (100 nM) or PDGF-BB (20 ng/mL) and were then incubated at 37°C for 24 h. The cells were fixed with methanol. The migration activity of the cells was expressed as the number of cells that migrated into the wound area in each field.

### Adenovirus Expression Vector and Plasmid Constructs

Adenovirus vector encoding circ-Sirt1 (Ad-circ-Sirt1) and GFP control (Ad-Vector) were entrusted to Hanbio shanghai. The expression plasmid of circ-Sirt1 was created by the placement of mouse entire circ-Sirt1 sequence into pcDNA3.1 circRNA Mini Vector (Addgene). The sequence of circ-Sirt1 mutated four of p53 binding regions were synthesized and inserted into pcDNA3.1 circRNA Mini Vector to overexpress mutant circ-Sirt1.

### Small Interfering RNA (SiRNA) Transfection

The siRNA duplexes targeting mouse circ-Sirt1 (si-circ-Sirt1-1 and si-circ-Sirt1-2) were obtained from GenePharma. Scrambled siRNA (si-Con) served as a negative control. The siRNAs were transiently transfected into VSMCs using Lipofectamine® RNAiMAX Transfection Reagent (Invitrogen) according to the manufacturer's protocol.

### Western Blot Analysis

RIPA buffer was used to lyse cells (50 mM Tris-Cl, pH 7.5, 1% NP-40, 0.5% Na-deoxycholate, 150 mM NaCl supplemented with complete proteinase inhibitor, Roche Applied Sciences) and mice carotid arteries (50 mM Tris-Cl, pH 7.5, 1% NP-40, 0.5% Na-deoxycholate, 0.1% SDS, 1 mM EDTA, 150 mM NaCl supplemented with complete proteinase inhibitor). Equal amounts of protein (30–60 μg) were separated by 10% SDS-PAGE, and electrotransfered to a PVDF membrane. Membranes were blocked with 5% milk in TBS for 1 h at room temperature, and incubated with primary antibodies against p53 (1:500, Abcam), p21 (1:200, Abcam), PCNA (1:1000, Santa Cruz), γ-H2AX (1:500, Abcam), GAPDH (1:1000, Cell Signaling Technology).

### RNA Extraction and Quantitative Reverse Transcription-PCR (qRT-PCR)

Total RNAs from cell lysates and tissues were isolated using TRIzol reagent (Life Technologies). The nuclear and cytoplasmic fractions were extracted using Minute TM Cytoplasmic and Nuclear Extraction Kit (Invent Biotechnologies). To quantify the amount of mRNA and circRNA, cDNAs were synthesized using the M-MLV First Strand Kit (Life Technologies), and quantitative PCRs were performed using SYBR Green qPCR SuperMix-UDG (Life Technologies). For quantification, all RNA expression was normalized to the amount of GAPDH using the 2^−Δ*ΔCt*^ method. The sequence for each primer was listed in Supplementary Table 1.

### Fluorescence *in situ* Hybridization (FISH)

Vascular tissues were fixed in 4% paraformaldehyde for 1 h and washed in DEPC-treated PBS, and then embedded in paraffin and serially sectioned for histological staining (10 μm thick). VSMCs grown on sterile glass coverslips were fixed with 4% paraformaldehyde for 30 min and permeabilized overnight in 70% ethanol.

For FISH, tissue section or cell coverslips were incubated with specific probe of 5' labeled by Biotin according to user manual of RNA FISH Kit SA-Biotin SYSTEM (GenePharma). NC and 18S-probe used as negative and positive control designed and synthesized by GenePharma, respectively. After stringent washing with elution buffer, cell nuclei were counterstained with DAPI (Invitrogen). Images were acquired using a Leica microscope (Leica SP5, Switzerland) and digitized with a software program LAS AF Lite.

### Immunofluorescence Staining

Carotid arteries were fixed with 4% paraformaldehyde for 1 h, washed in PBS, and then embedded in optimum cutting temperature (OCT) without further dissection. Serial sections (5 μm thick) were obtained at 500 μm proximal to the ligation site. VSMCs grown on sterile glass coverslips were fixed with 4% paraformaldehyde for 30 min, permeabilized with 0.1% Triton X-100 for 15 min.

For immunofluorescence, cells or tissues were blocked with 10% normal goat serum for 2 h. incubation with ACTA2 (1:200, Abcam), p53 (1:100, Abcam), NF-κB p65 (1:200, Cell Signaling Technology) in PBS at 4°Covernight. After washing three times in PBS, cells were incubated with secondary antibody (DyLight549 Conjugate; DyLight633 Conjugate, AmyJet Scientific) for 2 h at room temperature. Nuclei were counterstained with DAPI, and the coverslips were mounted on a glass slide. Confocal microscopy was performed with the Confocal Laser Scanning Microscope Systems (Leica).

### RNA Pull-Down Assay and Mass Spectrometry Analysis

Ad-circ-Sirt1-infected VSMCs were harvested after treatment with Ang II (100 nM) for 5 days. The cells were washed in ice-cold phosphate-buffered saline, lysed in 500 μL lysis buffer (20 mM Tris-HCl, pH 7.0, 150 mM NaCl, 0.5% NP-40, 5 mM EDTA, with freshly added 1 mM DTT, 1 mM PMSF, and 2 U/μL RNase inhibitor), and then incubated with 3 μg biotinylated DNA probe against circ-Sirt1 at 4 °C for 2 h, antisense biotinylated oligo probe as a control. A total of 50 μL Dynabeads™ MyOne™ Streptavidin C1 magnetic beads (Invitrogen) were added to each binding reaction and further incubated at 4 °C for 2 h. The beads were washed briefly with lysis buffer for three times. The bound proteins were analyzed by Western blot, or extracted and then analyzed by mass spectrometry. The results of mass spectrometry are present in Supplementary Table 2.

### RNA Immunoprecipitation Assay (RIP)

VSMCs were washed in ice-cold PBS, lysed in lysis buffer, and then used to conduct RIP experiments using a p53 antibody (Abcam) or IgG (Cell Signaling Technology), and the Dynabeads™ Protein G (Invitrogen) according to the manufacturer's instructions. The RNA fraction co-immunoprecipitated by anti-p53 antibody was extracted and quantified by NanoDrop 2000 (Thermo-Fisher). The cDNA was synthesized using a M-MLV First Strand Kit (Life Technologies) with random hexamer primers. The RIP circ-Sirt1 was subjected to qRT-PCR using the Platinum SYBR Green qPCR Super Mix UDG Kit (Invitrogen) and the ABI 7300 FAST system (Life Technologies).

### Chromatin Immunoprecipitation Assay (ChIP)

VSMCs were treated with 1–3% formaldehyde for 10 min to cross link proteins with DNA. The cross-linked chromatin was then prepared and sonicated to an average size of 500 bp. The samples were diluted 10-fold and then precleared with 10 μL Dynabeads™ Protein G (Invitrogen) for 30 min at 4°C. The DNA fragments were immunoprecipitated overnight at 4°C with the p53 (Abcam) or NF-κB p65 (Cell Signaling Technology) antibodies and normal mouse IgG (Cell Signaling Technology). The precipitated DNA was recovered *via* phenol/chloroform extraction, and the p53 binding site was amplified by qPCR.

### Statistical Analysis

All statistical analyses were performed with the SPSS 21.0 software. The data are presented as means ± SD. Two groups were compared by Student's *T*-tests. Differences among groups were analyzed with one-way analysis of variance (ANOVA). For all statistical comparisons, *P* < 0.05 was considered significant, and denoted with one and two asterisks when lower than 0.05 and 0.01, respectively.

## Results

### The Expression of circ-Sirt1 Is Decreased in the Arteries of Aged Mice and the Neointimal Hyperplasia

**Figure 1:**
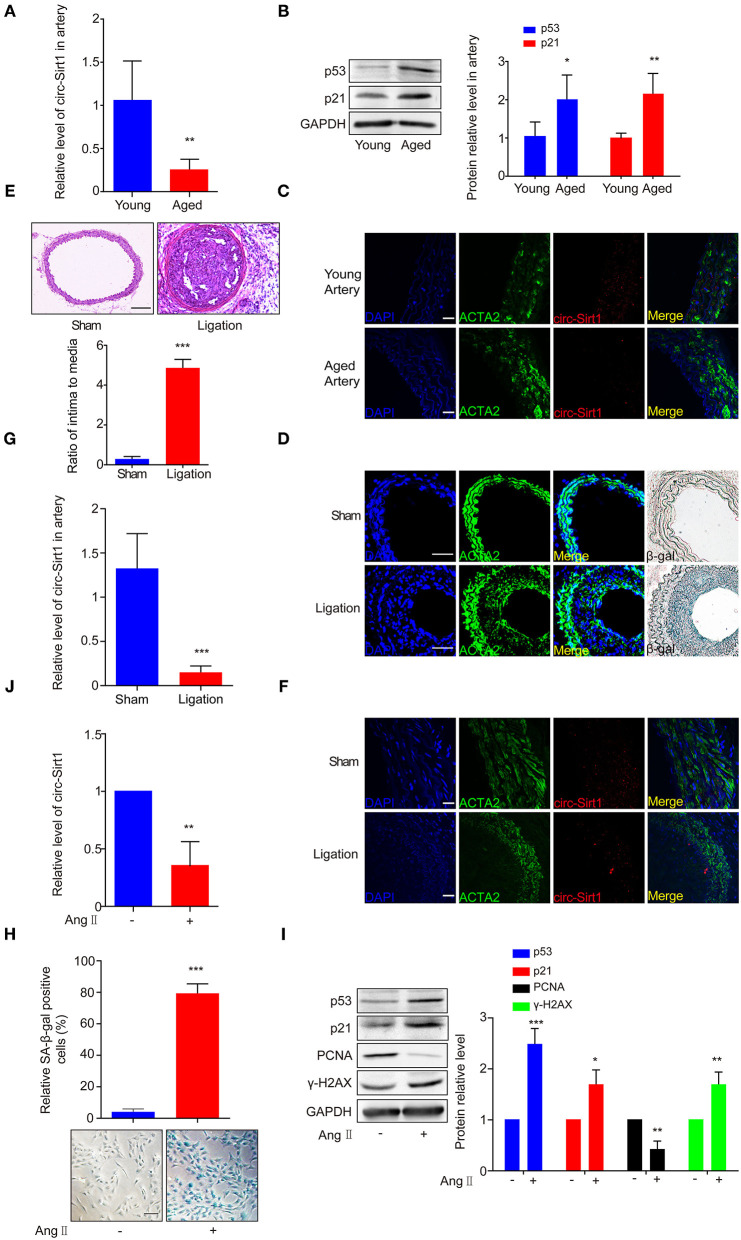
The expression of circ-Sirt1 is decreased in the arteries of aged mice and the neointimal hyperplasia. **(A–C)** qRT-PCR for circ-Sirt1 expression **(A)**, Western blot for p53 and p21 **(B)**, and representative co-localization analysis of circ-Sirt1 with the VSMC marker ACTA2 by fluorescent *in situ* hybridization in arteries of young and aged mice **(C)**. Scale bar = 25 μm. **(D)** Representative immunofluorescence for ACTA2 and SA-β-gal staining in the arteries at 14 days after ligation. Scale bar = 100 μm. **(E)** Representative hematoxylin and eosin-stained arterial sections from carotid arteries at 21 days after ligation. Scale bar = 100 μM. The ratio of intima to media (I/M). **(F,G)** Co-localization analysis of circ-Sirt1 with ACTA2 and qRT-PCRs for circ-Sirt1 expression in carotid arteries at 14 days after ligation. Scale bar = 25 μm. **(H–J)** Primary cultured VSMCs were stimulated with or without Ang II (100 nM) for 5 days. Representative senescence-associated β-galactosidase (SA-β-gal) staining for VSMCs **(H)**. Scale bar = 100 μm. Western blot for p53, p21, PCNA and γ-H2AX **(I)**. qRT-PCRs for circ-Sirt1 **(J)**. Bar graphs represent the mean ± SD and were analyzed with by Student's *T*-tests. *p < 0.05, **p < 0.01, ***p < 0.001 vs. control. *n* = 5/group for **(A,B,E,G)**; *n* = 3/group for **(H–J)**.

Based on the expression of circ-Sirt1 in mouse VSMCs (Supplementary Figure 1), to validate the association of circ-Sirt1 with vascular aging, we compared the level of circ-Sirt1 in the arteries of young and aged mice. We showed that the expression of circ-Sirt1 was decreased in the arteries of aged mice compared with young group (Figure 1A), accompanied with increased senescence markers p53 and p21 levels (Figure 1B). By RNA *in situ* hybridization, we further confirmed this reduction (Supplementary Figure 2 and Figure 1C). Our recent study demonstrated that the expression of circ-Sirt1 was reduced in neointimal formation (8). To determine the relationship between circ-Sirt1 and VSMC senescence in neointimal hyperplasia, we then established carotid artery ligation injury model in mice. Using SA-β-gal staining, we found that SA-β-gal positive cells markedly increased in neointimal formation (Figure 1D) with increased intima to media ratio (I/M) (Figure 1E). Furthermore, most of these cells were smooth muscle marker ACTA2-positive VSMCs, which mainly located in the neointima region and rarely in the media (Figure 1D). qRT-PCR and RNA *in situ* hybridization verified that circ-Sirt1 was decreased in neointimal formation (Figures 1F,G), indicating that VSMC senescence may be correlated with reduced circ-Sirt1 production.

To further confirm the relationship between circ-Sirt1 and senescence, VSMCs isolated from mouse aorta were treated with Ang II (100 nM) for 5 days to induced a senescence phenotype (17). After chronic treatment with Ang II, VSMCs displayed an enlarged and flattened morphology (Supplementary Figure 3). SA-β-gal-positive cells were significantly increased, accompanied with increased p53, p21, and γ-H2AX expression (Figures 1H,I) and decreased level of PCNA (proliferating cell nuclear antigen, a marker of cell growth) (Figure 1I). The expression of circ-Sirt1 was decreased, coincident with increased cellular senescence (Figure 1J). Taken together, these results suggest that decrease in circ-Sirt1 expression may be responsible for VSMC senescence in neointimal formation.

### circ-Sirt1 Inhibits Ang II-Induced Senescence

**Figure 2:**
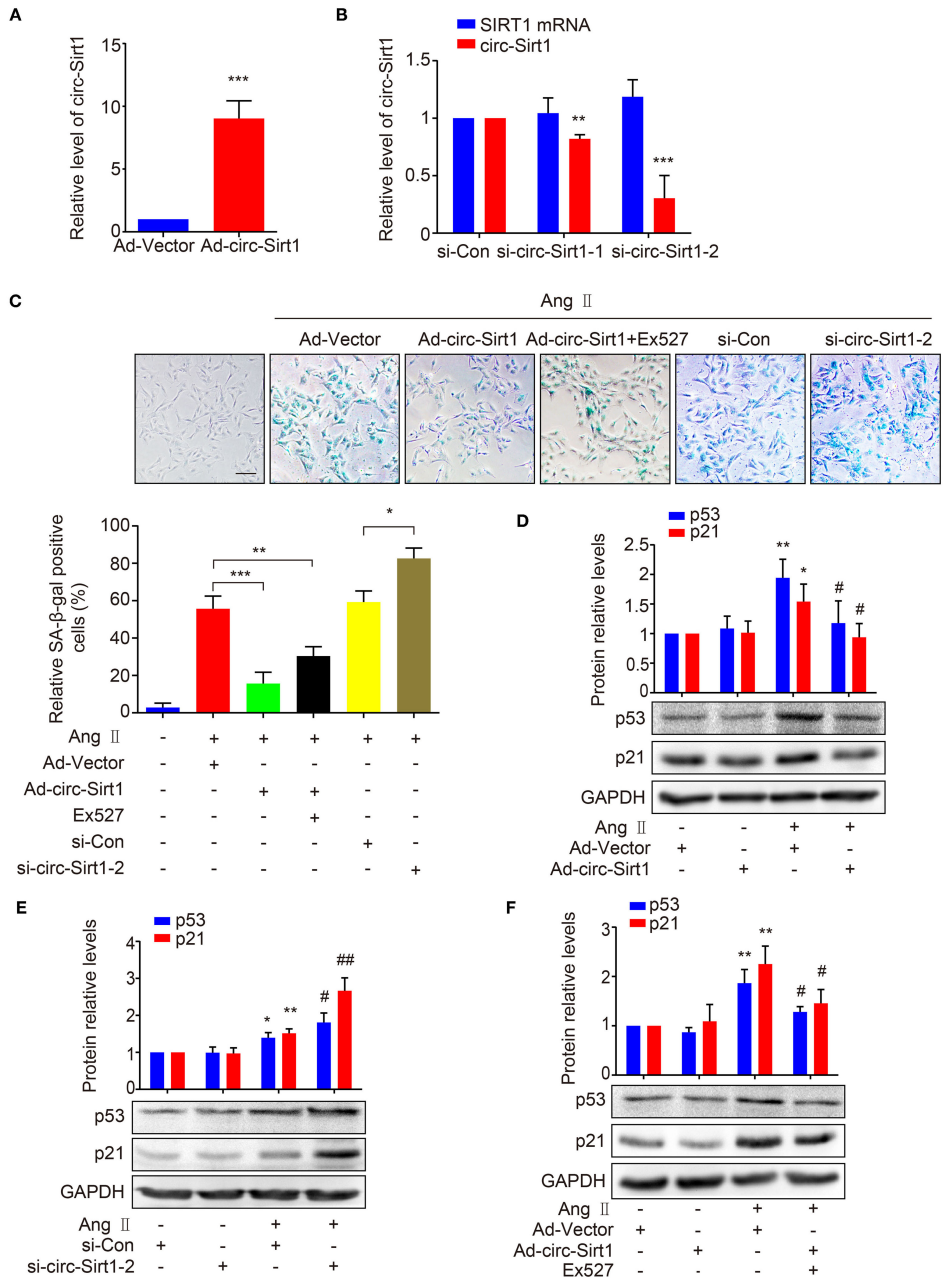
circ-Sirt1 inhibits Ang II-induced senescence. **(A,B)** The expression of circ-Sirt1 increased in Ad-circ-Sirt1-infected VSMCs **(A)**, and decreased in VSMCs treated with si-circ-Sirt1 **(B)**. **(C)** VSMCs were infected with Ad-Vector or Ad-circ-Sirt1 and then treated with or without Ang II (100 nM) for 5 days. For inhibition of SIRT1 activity, the cells were pretreated with Ex527 (20 μM) for 4 h before treated with Ang II. Representative senescence-associated β-galactosidase (SA-β-gal) staining. **(D–F)** Western blot for expression of p53 and p21. Bar graphs represent the mean ± SD and were analyzed with Student's *T*-tests. *p < 0.05, **p < 0.01, ***p < 0.001 vs. Ad-Vector+Ang II untreated group. ^#^*p* < 0.05, ^##^*p* < 0.01 vs. Ad-Vector+Ang II group. *n* = 3/group for **(A–F)**.

To validate our speculation, we examined the effect of circ-Sirt1 overexpression on Ang II-induced VSMC senescence. We showed that circ-Sirt1 expression was significantly increased in VSMCs infected with Ad-circ-Sirt1 and decreased in VSMCs treated with specific siRNA targeting the back-splice sequence of circ-Sirt1 (Figures 2A,B). Overexpression of circ-Sirt1 decreased the number of SA-β-gal-positive cells in Ang II-treated VSMCs (Figure 2C), accompanied by reduced expression of senescence markers p53 and p21 (Figure 2D). Similar change was observed in PDGF-BB-treated VSMCs (Supplementary Figure 4). Except for inducing senescence, Ang II and PDGF-BB also can promote the proliferation and migration of VSMCs. To investigate the impact of circ-Sirt1 on VSMC migration, we performed a cell-wounding assay, and showed that the migration was attenuated in Ad-circ-Sirt1-infected VSMCs upon stimulation with Ang II or PDGF-BB for 24 h (Supplementary Figure 5). In contrast, knockdown of endogenous circ-Sirt1 resulted in increased positive SA-β-gal staining and expression of p53 and p21 induced by Ang II (Figures 2C,E). Previous studies have demonstrated that SIRT1 inactivates p53 by deacetylation to inhibit cellular senescence (18, 19). Using Western blot, we determined that circ-Sirt1 promoted the expression of SIRT1 protein and reduced the level of p53 acetylation in Ang II-treated VSMCs (Supplementary Figure 6). To confirm whether the inhibitory effect of circ-Sirt1 on VSMC senescence completely depends on SIRT1, we used a specific SIRT1 inhibitor Ex527 to inactivate SIRT1 in Ad-circ-Sirt1-infected VSMCs. The result showed that Ex527 could not completely eliminate anti-senescence of circ-Sirt1 (Figures 2C,F). Overall, these data suggest that circ-Sirt1 inhibits p53-mediated senescence partly in SIRT1-dependent manner in VSMCs.

### circ-Sirt1 Interacts With and Anchors p53 in the Cytoplasm

**Figure 3:**
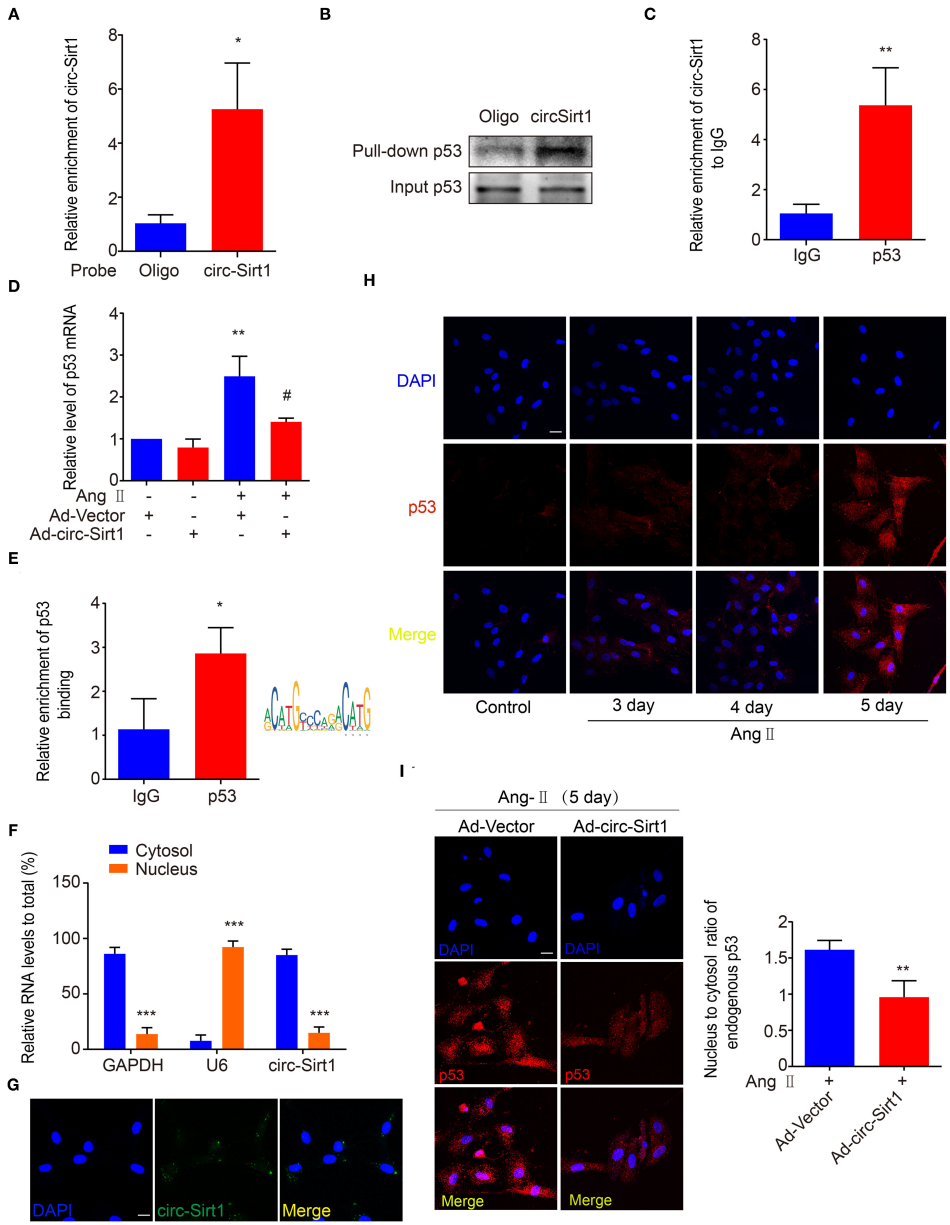
circ-Sirt1 interacts with and anchors p53 in the cytoplasm. **(A)** The probe specifically bound to circ-Sirt1. **(B,C)** RNA pull-down **(B)** and Rip **(C)** assays were performed to validate the interactions between circ-Sirt1 and p53 in senescent VSMCs. **(D)** The mRNA level of p53 decreased in senescent VSMCs infected with Ad-circ-Sirt1. Bar graphs represent the mean ± SD and were analyzed with Student's *T*-tests. **p < 0.01 vs. Ad-Vector+Ang II untreated group. ^#^*p* < 0.05 vs. Ad-Vector+Ang II group. **(E)** ChIP assay for p53 bound to p53 DNA elements in VSMCs treated with Ang II. **(F)** qRT-PCRs for circ-Sirt1 expression in the nuclear and cytoplasm fractions of VSMCs. **(G)** RNA *in situ* hybridization for the localization of circ-Sirt1 in VSMCs. Scale bars = 25 μm. **(H)** Representative immunofluorescence showing the localization of p53 in Ang II-treated VSMCs at 3, 4, and 5 days. Scale bars = 25 μm. **(I)** Overexpressing circ-Sirt1 decreased nuclear translocation of p53 in VSMCs treated with Ang II. Scale bars = 25 μm. Bar graphs represent the mean ± SD and were analyzed with Student's *T*-tests. *p < 0.05, **p < 0.01, ***p < 0.001 vs. control. *n* = 3/group for **(A–H)**. *n* = 12/group for **(I)**.

More recently, accumulated researches have demonstrated that circRNAs bind and sequester specific proteins to appropriate subcellular positions, and participate in modulating certain protein-protein and protein-RNA interactions (20). To investigate whether there is the SIRT1-independent mechanism underlying circ-Sirt1 inhibiting senescence, we attempted to identify the proteins that interact with circ-Sirt1 in Ang II-induced senescent VSMCs. A biotinylated DNA probe targeting the circ-Sirt1 back-spliced sequence was used to perform RNA pull-down assays, antisense biotinylated oligo probe as a control and mass spectrometry analysis to screen circ-Sirt1-interacting proteins. We determined that the DNA probe was specific and effective for circ-Sirt1 detection (Figure 3A). Removal of non-specific proteins bound by antisense oligo probe, only 29 proteins specifically interacting with circ-Sirt1 were identified in the total mass spectrometry results (Supplementary Table 2). Next, we used GO enrichment analysis to cluster and characterize these proteins according to their biological processes (Supplementary Figure 7). Coincidentally, p53 was also selected as a candidate for its important role in cellular senescence through induction of cell cycle arrest (Supplementary Figure 7). We then confirmed the interaction between circ-Sirt1 and p53 using RNA pull-down followed by Western blot and RIP assay (Figures 3B,C).

As above mentioned, overexpression of circ-Sirt1 significantly decreased the p53 protein level in Ang II-treated VSMCs (Figure 2C). We further determined that the expression of p53 mRNA was reduced when circ-Sirt1 was overexpressed (Figure 3D). Studies in tumor-derived cells have demonstrated that p53 protein directly binds to its own promoter to induce the transcription of itself and downstream target genes responsible for cell cycle arrest and DNA repair (21, 22). Therefore, we predicted *p53* responsive element and confirmed the DNA binding for p53 transcriptional autoactivation in senescent VSMCs using ChIP (Figure 3E). These results lead us to speculate whether the interaction between circ-Sirt1 and p53 results in a cytoplasmic sequestration of p53, which in turn affects the expression of p53 and downstream target genes. To validate this hypothesis, we detected circ-Sirt1 expression in the cytoplasmic and nuclear fractions of VSMCs using qRT-PCR. We showed that circ-Sirt1 was primarily expressed in the cytoplasm of VSMCs (Figure 3F), and fluorescence *in situ* hybridization further verified this finding (Figure 3G). The expression of p53 was lower in quiescent VSMCs and remarkably increased upon chronic treatment with Ang II, accompanied by significant nuclear translocation (Figure 3H). However, circ-Sirt1 overexpression resulted in a reduced ratio of nucleus to cytoplasm under the same conditions (Figure 3I), indicating that cytoplasmic circ-Sirt1 may block p53 nuclear translocation *via* interaction between the two molecules. Taken together, these data suggest that circ-Sirt1 inhibits p53-mediated senescence in both direct (SRIT1-independent) and indirect (SIRT1-dependent) manners.

We recently demonstrated that circ-Sirt1 was involved in inhibiting NF-κB activation by direct interaction (8). To validate whether inhibiting NF-κB is important for circ-Sirt1 role in senescence, we tested the cellular distribution and activity of NF-κB p65. Immunofluorescence showed that nuclear translocation of NF-κB p65 was significantly increased after stimulation with Ang II for 24 h (Supplementary Figure 8A). Overexpression of circ-Sirt1 inhibited the Ang II-induced nuclear translocation of NF-κB p65 (Supplementary Figure 8B) and decreased its binding to the responsive element in the *p53* promoter, as determined by ChIP assay (Supplementary Figure 8C), suggesting that the inhibitory effect of circ-Sirt1 on VSMC senescence may at least partly involve in inhibiting NF-κB activation.

### Identification of circ-Sirt1 Binding to p53

**Figure 4:**
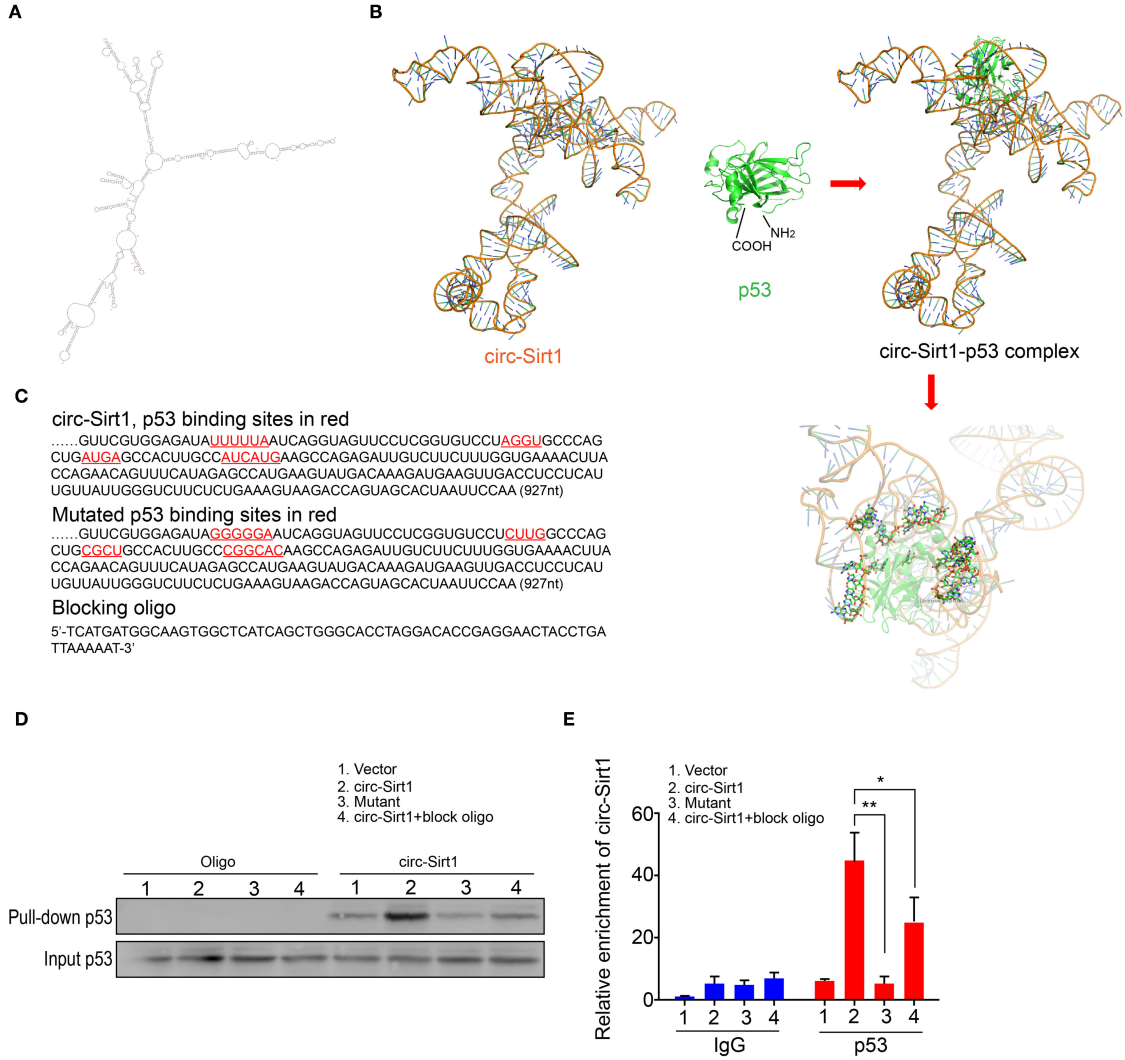
Identification of circ-Sirt1 binding to p53. **(A)** 2-D RNA structure of circ-Sirt1 was generated by Mfold. **(B)** Graphical representation of 3-D structures of circ-Sirt1 and p53 docking model with a zoom-in image of the binding interface done by NPDock. **(C)** Sequences of circ-Sirt1 for p53-binding sites within nt 729-792, designed mutated binding sites and blocking oligo. **(D)** RNA pull-down assays were performed in senescent VSMCs transfected with control vector, circ-Sirt1, mutant circ-Sirt1 and block oligo. **(E)** qRT-PCR showed that antibody against p53 pulled down more circ-Sirt1 from the circ-Sirt1-transfected cells than the control group, but not in the binding site mutated cells or cells treated with blocking oligo. Bar graphs represent the mean ± SD and were analyzed with Student's *T*-tests. *p < 0.05, **p < 0.01 vs. control vector. *n* = 3/group.

To identify the possible regions for the interaction of circ-Sirt1 with p53, we performed an algorithm analyses using Mfold that is a dynamic programming algorithm, which uses energy minimization to model the ensemble of possible structures by identifying optimal folding of a nucleic-acid sequence within a specified energy increment (23). The best predicted secondary structure of circ-Sirt1 was derived by analyzing its thermodynamic properties using the formula ΔG = ΔH-TΔS, ΔG = −223.76 kcal/mol at 37°C, ΔH = −278.97 kcal/mol, ΔS = −1492.1 cal/(Kmol), and generating RNA stem loop structures (Figure 4A). The secondary structure defined using dot-bracket notation was then analyzed by the software RNA composer for tertiary structure prediction (24). The structure of p53 protein used in the docking procedure was derived from Protein Data Bank (PDB) entry 2IOI. NPDock (25), a web server for predicting complexes of protein-nucleic acid structures, was then used to carry out the *in-silico* molecular docking between circ-Sirt1 and p53. The molecular simulation result supported that circ-Sirt1 could perfectly dock p53 and predicted a minimal binding region of circ-Sirt1 for the p53 within nt 729-792 (Figures 4B,C).

To validate the prediction, we constructed the circ-Sirt1 vector mutating p53 binding sites within binding region, synthesized blocking oligo that were complimentary to nt 729-792, and performed a pull-down experiment. We showed that the biotinylated probe pulled down significantly higher levels of p53 when the cells were overexpressed circ-Sirt1 (Figure 4D). But the interaction of p53 with circ-Sirt1 was weakened in VSMCs transfected with circ-Sirt1 mutant or blocking oligo (Figure 4D). We further verified the interaction by Rip assay. p53 antibody pulled down lower levels of circ-Sirt1 in mutant transfected VSMCs or when the cells were co-transfected with circ-Sirt1 and blocking oligo (Figure 4E). Collectively, these results further support that circ-Sirt1 directly binds to p53.

### Overexpression of circ-Sirt1 Inhibits Senescence-Related Neointimal Hyperplasia *in vivo*

**Figure 5:**
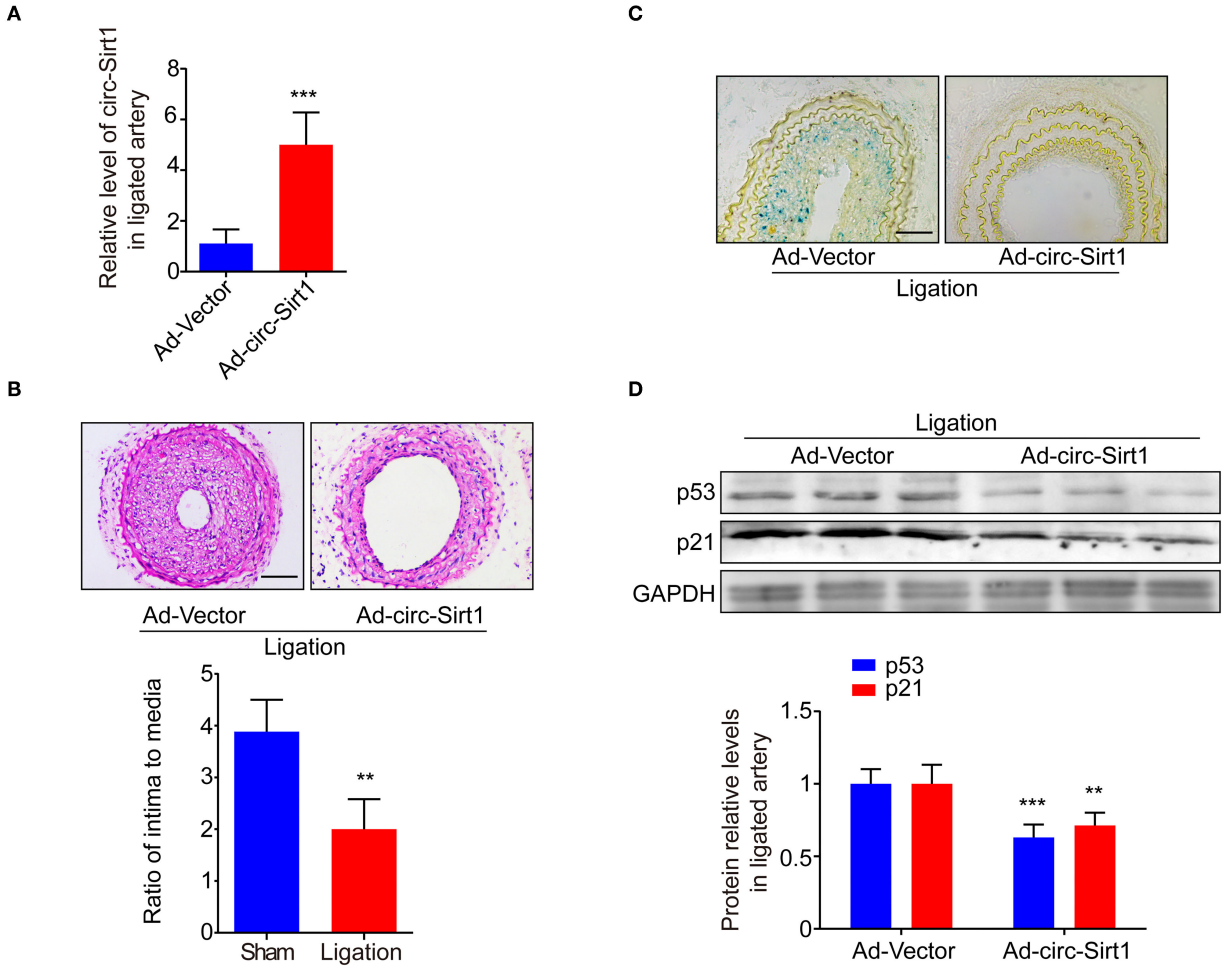
Overexpression of circ-sirt1 inhibits senescence-related neointimal hyperplasia *in vivo*. **(A)** qRT-PCRs for circ-Sirt1 in Ad-Vector or Ad-circ-Sirt1-infected carotid arteries at 14 days after ligation. **(B)** Representative hematoxylin and eosin-stained arterial sections from Ad-Vector or Ad-circ-Sirt1-infected carotid arteries at 14 days after ligation. Scale bar = 100 μM. The ratio of intima to media (I/M). **(C)** Representative senescence-associated β-galactosidase (SA-β-gal) staining. Scale bar = 50 μm. **(D)** Western blot for p53 and p21. Bar graphs represent the mean ± SD and were analyzed with Student's *T*-tests. **p < 0.01, ***p < 0.001 vs. Ad-Vector group. *n* = 5/group for **(A,B,D)**.

To explore whether the intervention of circ-Sirt1 can be a potential strategy for preventing neointimal hyperplasia by repressing senescence, an Ad-vector or Ad-circ-Sirt1 was infected into mice carotid arteries after ligation *in vivo*. We showed that circ-Sirt1 expression was significantly increased in carotid arteries infected with Ad-circ-Sirt1 (Figure 5A). Neointimal hyperplasia induced by injury was obviously repressed by overexpression of circ-Sirt1 (Figure 5B). Compared with the vehicle group, the I/M ratio was reduced in Ad-circ-Sirt1-infected neointima (Figure 5B), which further verified our previous finding (8). Importantly, the number of SA-β-gal-positive cells was decreased in Ad-circ-Sirt1-infected arteries compared to the vehicle control 14 days after ligation injury, accompanied by down-regulated expression of senescence markers p53 and p21 (Figures 5C,D).

To investigate whether the similar regulatory mechanism may involve in development of atherosclerosis, we constructed atherosclerotic model using ApoE^−/−^ mice. ApoE^−/−^ mice fed Paigen diet displayed prominent oil red O staining in the aortic sinus and aortic arch (Supplementary Figure 9A) and an aggravated atherosclerotic plaque lesion compared with WT mice (Supplementary Figure 9B). Furthermore, p53 positive cells significantly accumulated in the atherosclerotic lesions of ApoE^−/−^ mice, accompanied with increased SA-β-gal staining areas (Supplementary Figure 9C). Similar to the results observed in the neointima, the expression of circ-Sirt1 decreased in the atherosclerotic aortic tissues from ApoE^−/−^ mice compared with WT mice (Supplementary Figure 9D). In addition, we collected renal arterial specimens from patients with or without atherosclerosis or hypertension who underwent nephrectomy at the Fourth Hospital of Hebei Medical University, China. The atherosclerotic renal-artery wall exhibited neointimal hyperplasia. Immunofluorescence revealed that positive staining of p53 in human renal arterial neointima was higher than normal controls, accompanied with increased SA-β-gal activity (Supplementary Figure 9E). The circ-Sirt1 level was significantly decreased in the vascular tissue of patients with atherosclerosis or hypertension (Supplementary Figure 9F). Together, these findings suggest that impaired circ-sirt1 is involved in neointimal formation and atherosclerosis development *via* eliciting senescence. Targeting circ-sirt1 may be considered as a new therapeutic strategy for retarding aging-associated vascular diseases.

## Discussion

It is reported that many circRNAs exhibit additional regulatory functions in aging and aging-related diseases progression (26). The present study revealed that circ-Sirt1 was highly expressed in young and healthy arteries, and decreased in aged arteries and in the neointima and atherosclerotic lesion. Overexpression or knockdown of circ-Sirt1 delayed or accelerated Ang II-induced VSMC senescence *in vitro* and injury-induced neointimal hyperplasia *in vivo via* modulation of p53 activity in SIRT1-dependent and SIRT1-independnet manners. Thus, circ-Sirt1 ameliorates aging-related vascular diseases.

Growing evidences suggest that multiple non-coding RNAs are involved in regulating VSMC senescence (27), and are becoming master regulators of senescent signaling by fine-tune p53 activity (28, 29). Unlike miRNAs directly targeting key proteins in p53 signaling pathway, circRNAs play their functional role through acting as miRNA and protein sponges or facilitating contact and assembly of proteins. Previous studies indicated that circRNAs such as circFoxo3 (30), circ-Ccnb1 (31), and circ-DNMT1 (32) involved in regulating p53 signal *via* inducing p53 proteasomal degradation by binding to p53 and MDM2, linking the interaction of H2AX and wild-type p53 thus allowing cell survival or promoting Dnmt1 expression and nuclear translocation to inhibit p53 transcription. Our group has also demonstrated that accumulation of SM22α that is actin binding protein, accelerates senescence of vascular smooth muscle cells *via* stabilization of p53 *in vitro* and *in vivo* (17). However, little is known about the role and regulation of circRNAs in p53 activation and the relationship with VSMC senescence. The present study determined that circ-Sirt1 inhibits p53 expression *via* interacting with p53 to block p53 nuclear translocation and SIRT1-mediated p53 deacetylation and inactivation. Furthermore, we characterized the possible binding region of circ-Sirt1 with p53, and showed that mutated circ-Sirt1 restrained the interaction of circ-Sirt1 with p53. Our findings suggest that circ-Sirt1 may be a new endogenous circRNA inhibitor of p53 signaling. As a key regulator of cellular senescence, p53 is the first non-histone substrates identified to be functionally associated with the anti-senescence activity of SIRT1 (33). Previous study suggested that SIRT1 confers VSMC resistance to senescence partially *via* enhancing p53 deacetylation (34). The current study found that overexpression of circ-Sirt1 promoted SIRT1 protein expression and reduced p53 acetylation during Ang II-induced senescence of VSMCs.

VSMCs, as basic ingredients of the vascular wall and the sole cell type in the arterial medial layer, play a pivotal role in physiological and pathological conditions. Studies in both humans and animals have indicated that senescent VSMCs were increased in atherosclerotic lesions, including fatty streaks and plaques (35). Neointimal formation mediated by the proliferation and migration of VSMCs is a common pathological basis for atherosclerosis and restenosis (36). Aging also induces VSMC phenotypic modulation that could influence on cell senescence and loss of plasticity and reprogramming (37). VSMC senescence *in vivo* is likely to be driven by multiple pathways, including DNA damage, mitochondrial deterioration and oxidative stress, which are all present during atherosclerosis. Replicative senescence is highly relevant in the context of neointima and atherosclerotic plaque VSMC proliferation, because the generation of all the VSMC-derived cells by proliferation would probably cause replicative senescence (9). This may explain why cellular senescence is mainly restricted to the intimal area. In keeping with this idea, the telomeres of VSMCs in human atherosclerotic plaques are markedly shortened, which correlates with disease severity (38). In the present study, we demonstrated that disruption of circ-Sirt1 is associated with increased neointimal formation and VSMC senescence. Overexpression of circ-Sirt1 inhibited AngII-induced VSMC senescence. Interestingly, positive SA-β-gal staining mainly appeared in the neointima, rather than in the media, consistent with finding of previous study (39). This phenomenon can be attributed to the greater proliferation ability of dedifferentiated VSMCs in the neointima, as repeated cell division leads to critical shortening and erosion of the telomeres and loss of the protective shelterin complex, which results in a persistent DNA damage response (DDR) that instigates senescence (9). Contradictively, another study showed increased SA-β-gal areas in both media and neointima (40). In addition, we found increased p53 positive cells and decreased circ-Sirt1 expression within atherosclerotic lesion of ApoE^−/−^ mice fed with Paigen diet compared with WT mice. Similarly, the same results were observed in renal arterial specimens from patients with atherosclerosis. Thus, these results suggest that disruption of circ-Sirt1 may involve in development of atherosclerosis.

NF-κB nuclear translocation and activation are a prerequisite for the expression of inflammatory related gene. It was also proposed that p53 and NF-κB signaling pathways antagonize each other by a negative regulatory loop (41, 42). Moreover, some reports indicated that p53 could activate inflammatory responses in NF-κB-independent manner (43, 44) and the feedback inhibitory effect of p53 on NF-κB during senescence (41, 42). In the present study, we showed that overexpression of circ-Sirt1 inhibited the Ang II-induced nuclear translocation of NF-κB p65 and decreased its binding to the responsive element in the *p53* promoter. Since both p53 and NF-κB are the inhibitory targets downstream of circ-Sirt1, together intimate association between inflammation and senescence, it is important to explore the spatiotemporal relationship of p53 and NF-κB in the pathway of circ-Sirt1 inhibiting senescence in future study. In addition, we could not exclude the possibility that the contribution of VSMCs to senescence is underestimated or overestimated in neointimal formation, as macrophages are observed to express SMC markers in human coronary atherosclerosis (45), while many SMC-derived cells within advanced lesions of ApoE^−/−^ mice lack detectable expression of conventional SMC markers such as ACTA2 (46). The other critical challenge for future study will be to fully characterize which subset of VSMC are becoming senescent and losing circ-Sirt1 using a lineage tracing approach.

## Data Availability Statement

The original contributions presented in the study are included in the article/Supplementary Material, further inquiries can be directed to the corresponding authors.

## Ethics Statement

The studies involving human participants were reviewed and approved by The Ethical Committee of Hebei Medical University. The patients/participants provided their written informed consent to participate in this study. The animal study was reviewed and approved by the Hebei Medical University Institutional Animal Care and Use Committee.

## Author Contributions

PK, C-LL, and Y-QD conducted experiments, analyzed data, generated the illustrations, and wrote the manuscript. LC and X-YZ conducted *in vivo* experiments. W-DZ, Z-QB, Z-YP, and A-QY performed *in vitro* experiments. MH supervised the project and edited the manuscript. All authors read and approved the final manuscript.

## Funding

This work was supported by the National Natural Science Foundation of China (91849102 and 91739301); the Key Natural Science Foundation Projects of Hebei Province (H2019206028); Natural Science Foundation of Hebei Province (H2021206006 and H2021423047); Natural Science Youth Fund of Hebei Province Education Department (QN2020167); and Medical Science Research Subject Project of Hebei Province (20222400).

## Conflict of Interest

The authors declare that the research was conducted in the absence of any commercial or financial relationships that could be construed as a potential conflict of interest.

## Publisher's Note

All claims expressed in this article are solely those of the authors and do not necessarily represent those of their affiliated organizations, or those of the publisher, the editors and the reviewers. Any product that may be evaluated in this article, or claim that may be made by its manufacturer, is not guaranteed or endorsed by the publisher.
