# Supplementary material for: circ-Sirt1 Decelerates Senescence by Inhibiting p53 Activation in Vascular Smooth Muscle Cells, Ameliorating Neointima Formation
Source: Front Cardiovasc Med. 2021 Dec 17;8:724592. doi: 10.3389/fcvm.2021.724592 (PMC8718546; doi:10.3389/fcvm.2021.724592)
Supplement: Supplementary file 1 [file Data_Sheet_1.docx]

Supplementary Material

**Supplementary Figure 1.** Determination of circ-Sirt1 expression in mice VSMCs. **（A, B）**RT-PCR product of full-length circ-Sirt1 in mice VSMCs was visualized by agarose gel electrophoresis (A), and sequenced to confirm circ-Sirt1 junction sequences (B).

**Supplementary Figure 2.** RNA-FISH assays were conducted to detect circ-Sirt1 in mouse artery using biotin-labeled sense and scramble control (NC). Nuclei were stained with DAPI. Scale bar=25 μm.

**Supplementary Figure 3.** Ang II induced senescent VSMCs display larger and flattened morphology. VSMCs treated with Ang Ⅱ (100 nM) for 5 days. Scale bar=100 μm. ^***^*p*<0.001 versus Ang Ⅱ untreated group. n=30/group.

**Supplementary Figure 4**. circ-Sirt1 inhibits PDGF-BB-induced senescence. VSMCs were infected with Ad-Vector or Ad-circ-Sirt1 and then treated with or without PDGF-BB (20 ng/mL) for 3 days. Representative senescence-associated β-galactosidase (SA-β-gal) staining. Scale bar=100 μm. Bar graphs represent the mean±SD and were analyzed with Student's T tests. ^***^*p*<0.001 versus Ad-Vector+PDGF-BB untreated group. ^##^*p*<0.01, versus Ad-Vector+PDGF-BB group. n=3/group.

**Supplementary Figure 5**. The effect of circ-Sirt1 on VSMC migration. **(A, B)** The relative activity of migration using a cell-wounding assay. Scale bar=200 μm. Bar graphs represent the mean±SD and were analyzed with Student's T tests. ^*^*p*<0.05, ^**^*p*<0.01 versus Ad-Vector, Ad-Vector+Ang II or Ad-Vector+PDGF-BB group. n=3/group.

**Supplementary Figure 6.** circ-Sirt1 deacetylates p53 via promotion of SIRT1 expression. VSMCs infected with Ad-Vector or Ad-circ-Sirt1 following treated with or without Ang II (100 nM) for 2 h. Bar graphs represent the mean±SD and were analyzed with Student's T tests. ^**^*p*<0.01 versus Ad-Vector+Ang II untreated group. ^##^*p*<0.01 versus Ad-Vector+Ang II group. n=3/group.

**Supplementary Figure 7.** circ-Sirt1 interacts with and sequesters p53 in the cytoplasm. GO analysis annotates the biological process and clusters the modules of genes. The top 30 cluster ranked by *p* value was shown.

**Supplementary Figure 8.** circ-Sirt1 inhibits NF-κB p65 nuclear translocation and activation in Ang Ⅱ-treated VSMCs. **(A, B)** Representative immunofluorescence of NF-κB p65 in Ang Ⅱ-treated VSMCs (A), and overexpressing circ-Sirt1 decreased nuclear translocation of NF-κB p65 in VSMCs treated with Ang Ⅱ (100 nM) for 24 h (B). Scale bar=25 μm. **(C)** ChIP assay for NF-κB p65 binding to the DNA elements of p53 in VSMCs treated with Ang Ⅱ for 24 h after infected with Ad-Vector or Ad-circ-Sirt1. Bar graphs represent the mean±SD and were analyzed with Student's T tests. ^*^*p*<0.05 versus Ad-Vector+Ang II treated group. n=3/group.

**Supplementary Figure 9.** Impaired circ-Sirt1 expression is associated with development of atherosclerosis. **(A, B)** WT and ApoE^-/-^ mice fed Paigen diet for 8 weeks. Representative images of en face Oil Red O-stained aortas, aortic sinus and aortic arch sections (A) and quantification of lesion areas are shown (B). **(C, D)** Representative immunofluorescence and SA-β-gal staining (C), qRT-PCR for circ-Sirt1 expression (D) in the aortic sections from WT and ApoE^-/-^ mice fed Paigen diet for 8 weeks. Scale bar=100 μm. **(E)** Immunofluorescence and SA-β-gal staining in normal and neointimal hyperplasia human renal arteries. Scale bar=100 μm. **(F)** qRT-PCR for circ-Sirt1 expression in human renal arteries from patients with or without atherosclerosis or hypertension. Bar graphs represent the mean±SD and were analyzed with by Student's T tests. ^**^*p*<0.01, ^***^*p*<0.001 versus WT mice fed Paigen diet group. n=5/group for A, B, D, F.

Supplementary Figure 1


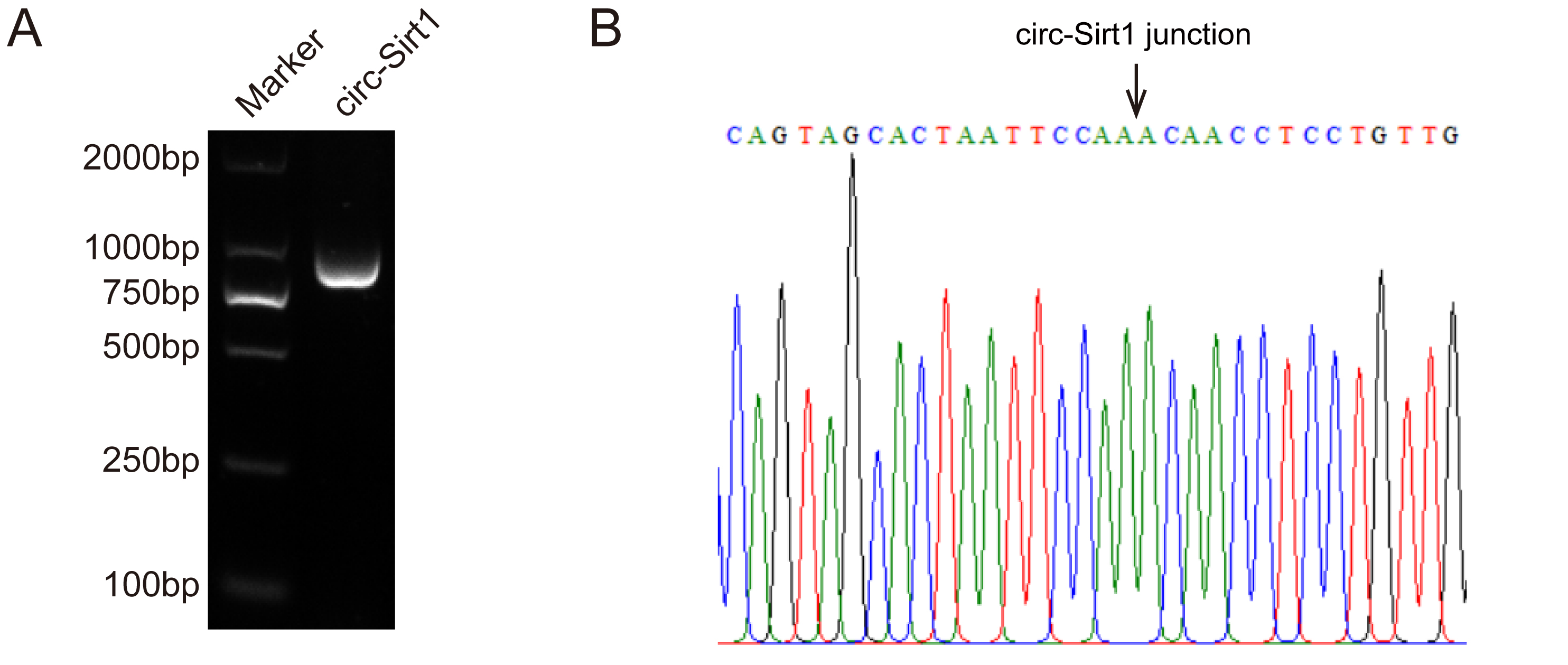


Supplementary Figure 2


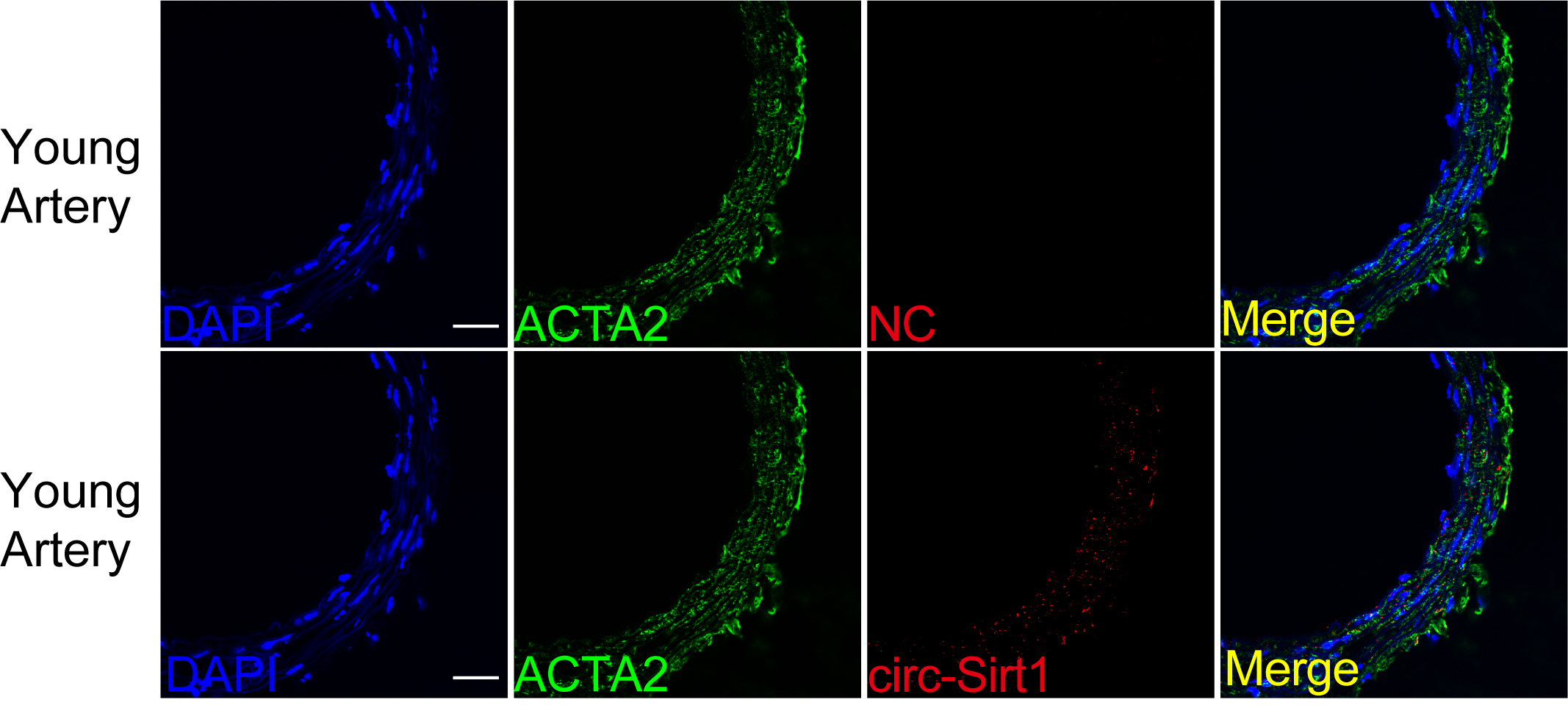


Supplementary Figure 3


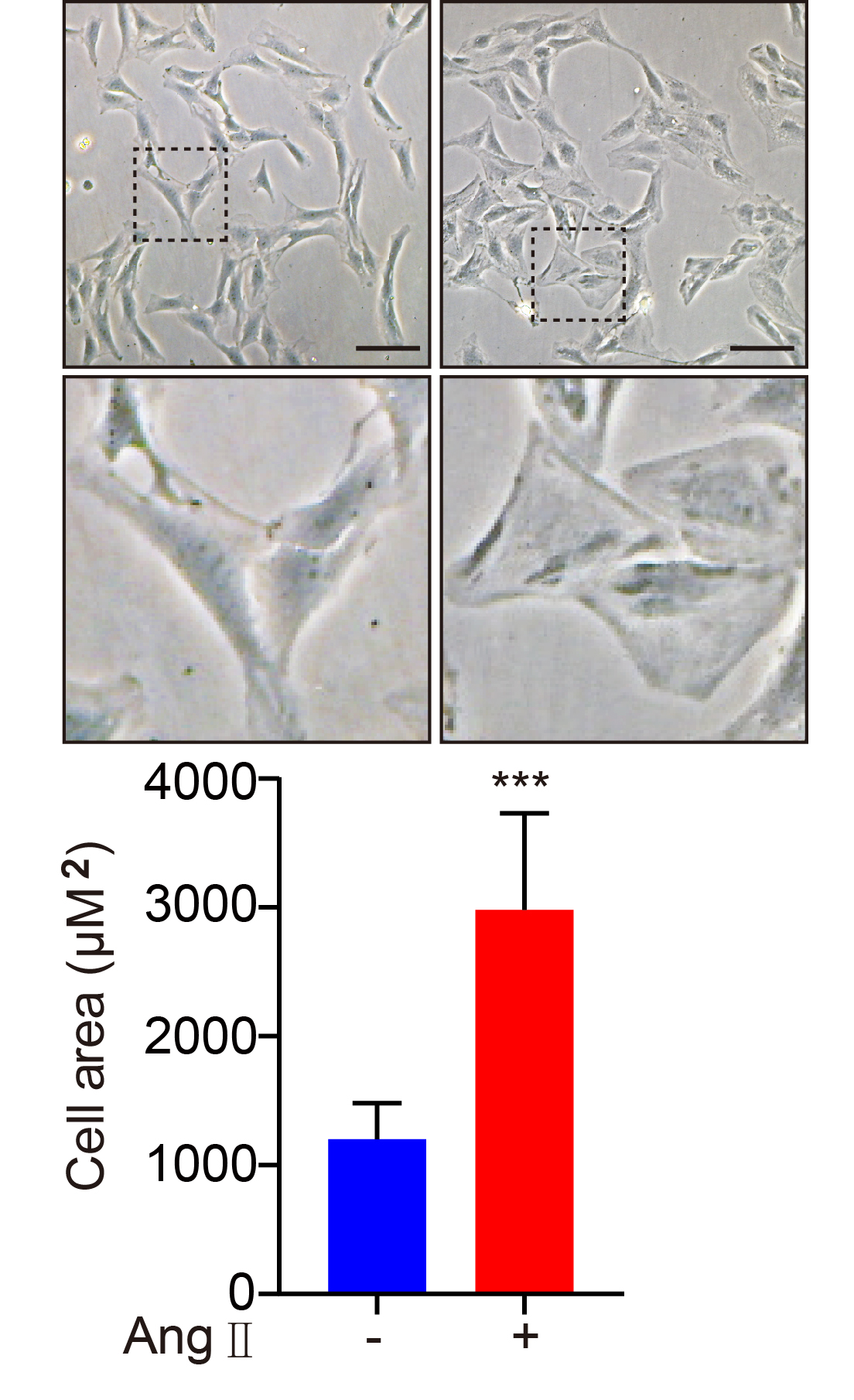


Supplementary Figure 4


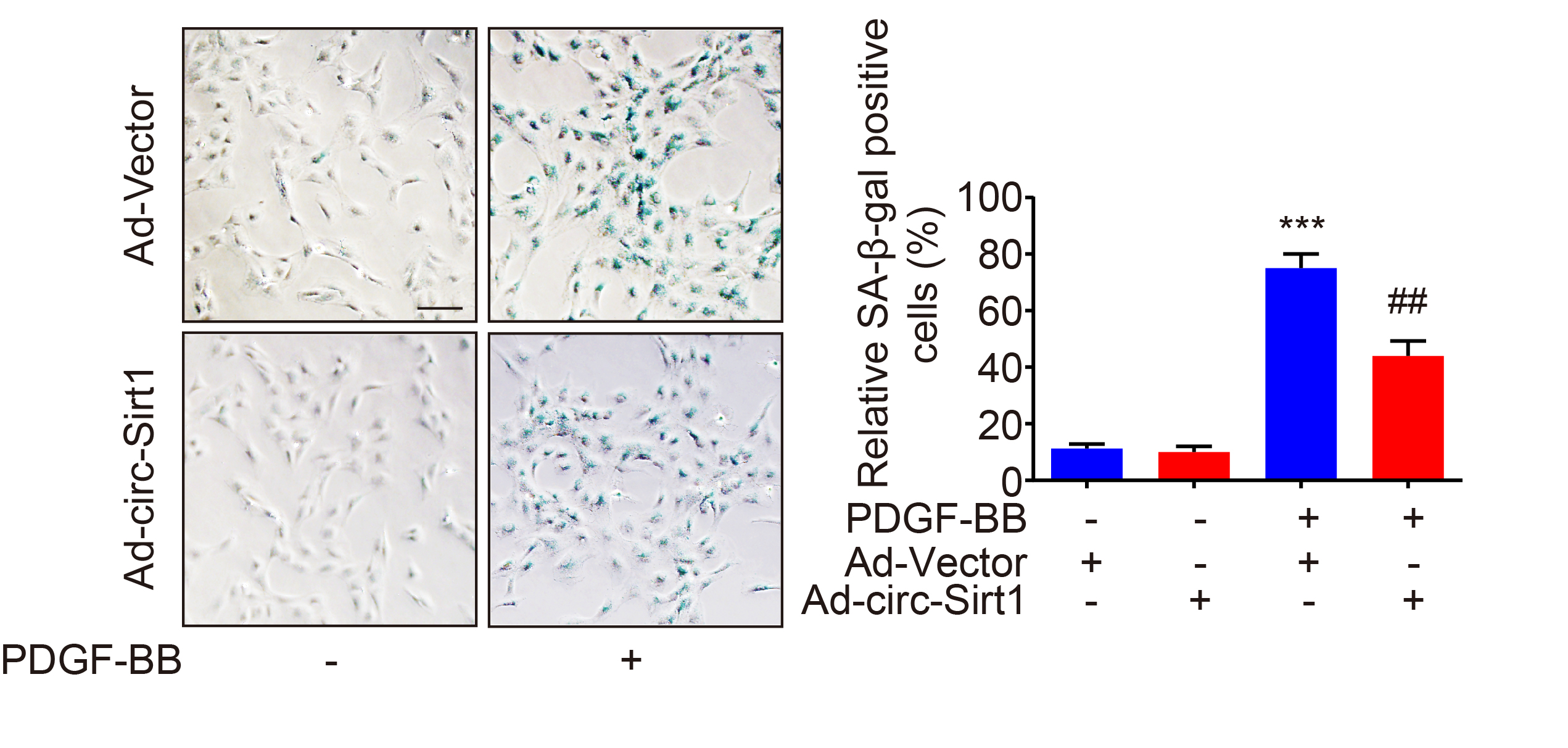


Supplementary Figure 5


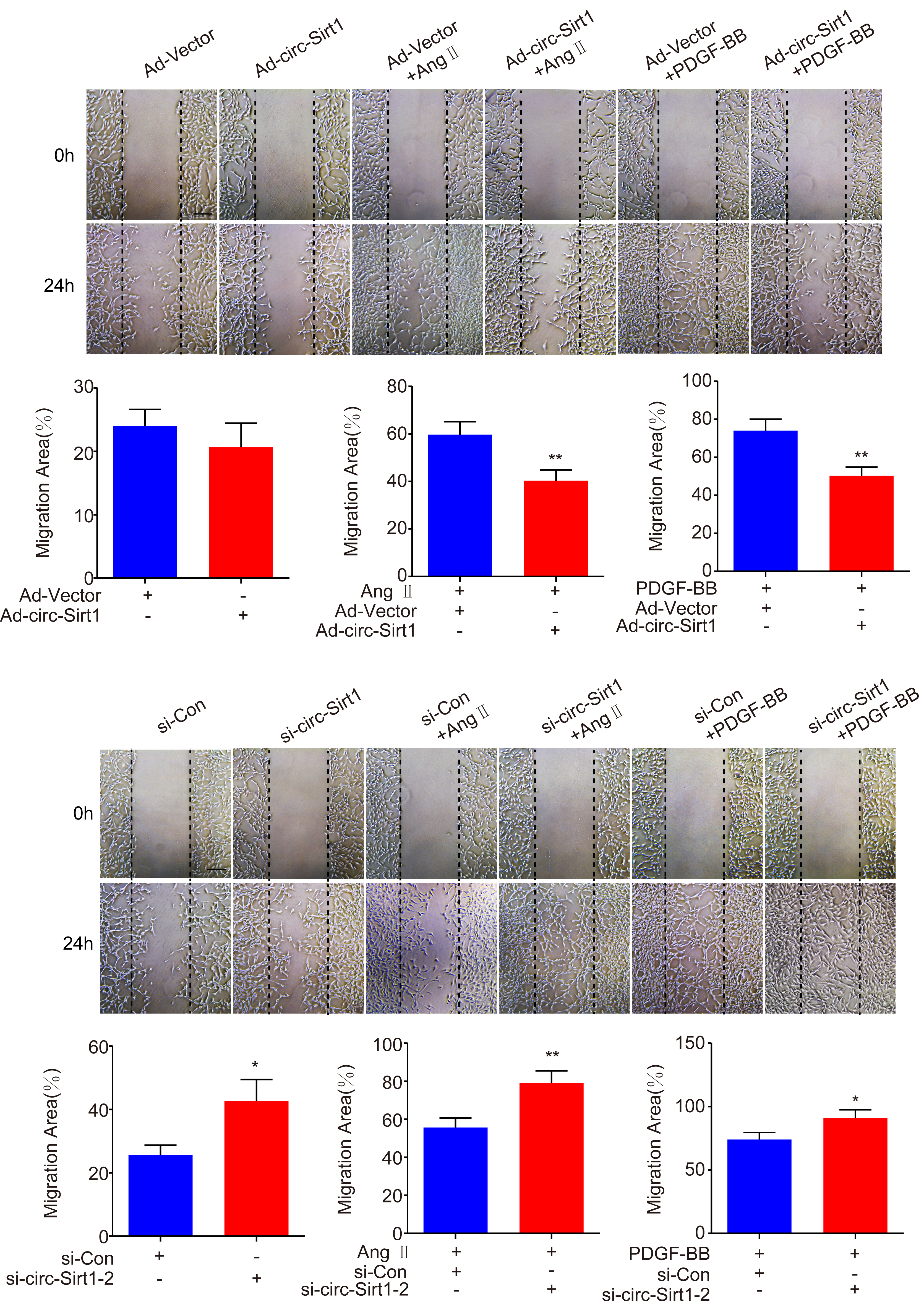


Supplementary Figure 6


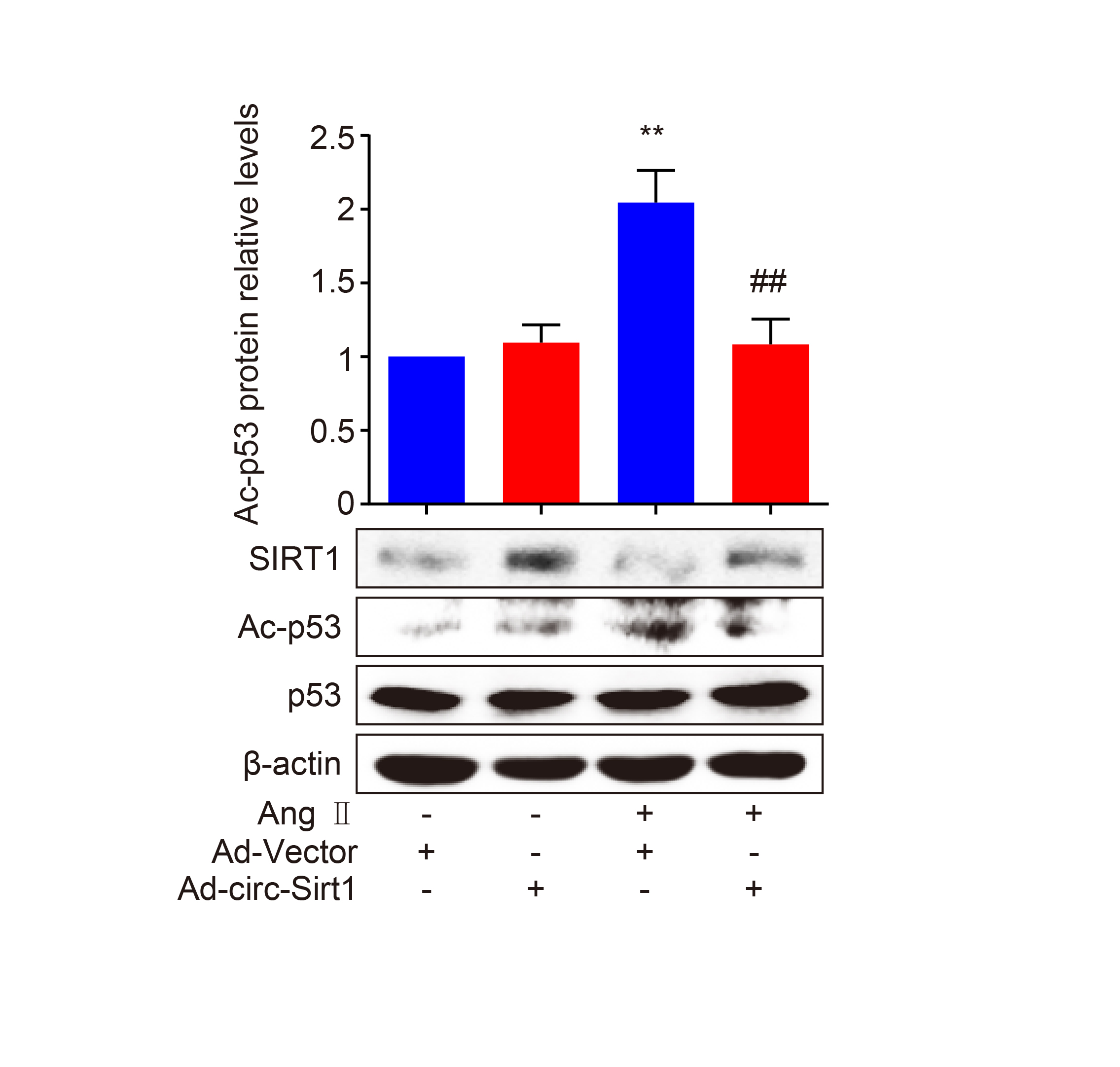


Supplementary Figure 7


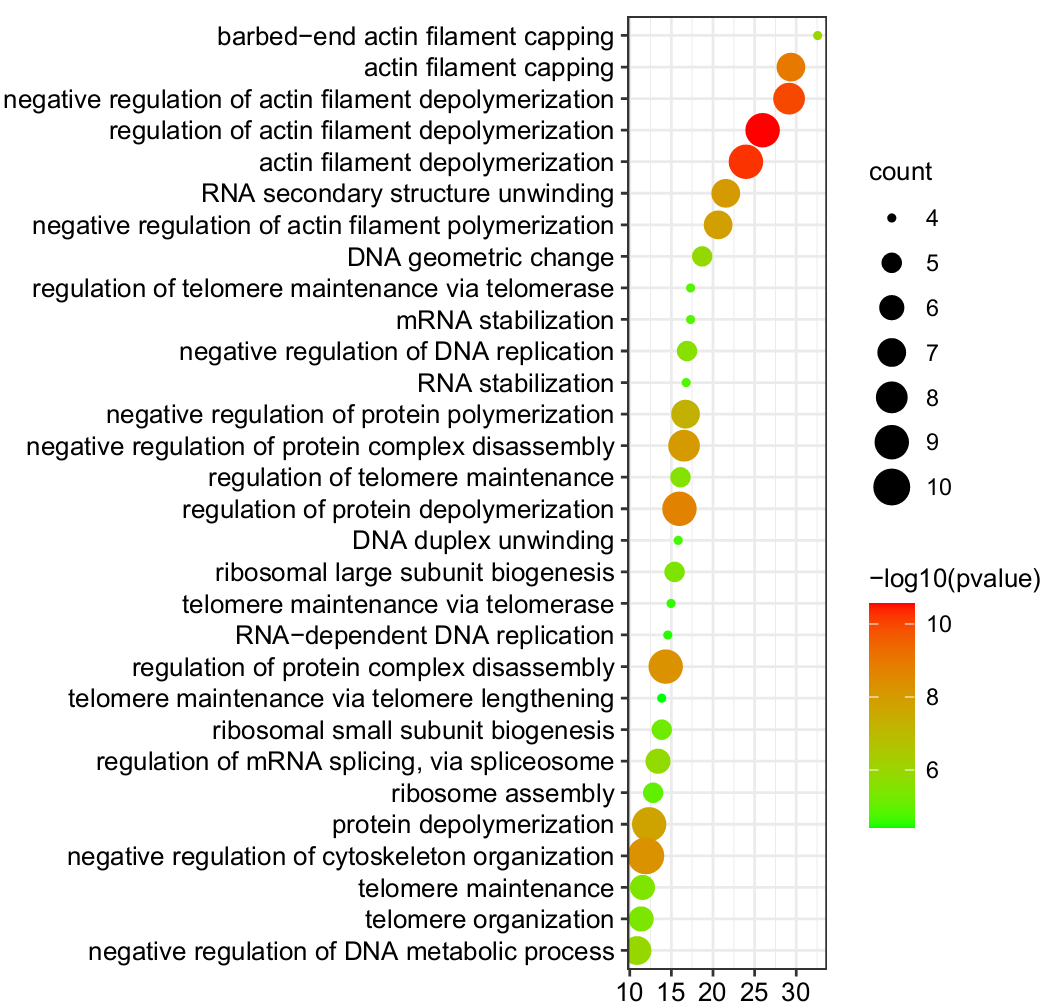


**Supplementary Figure 8**


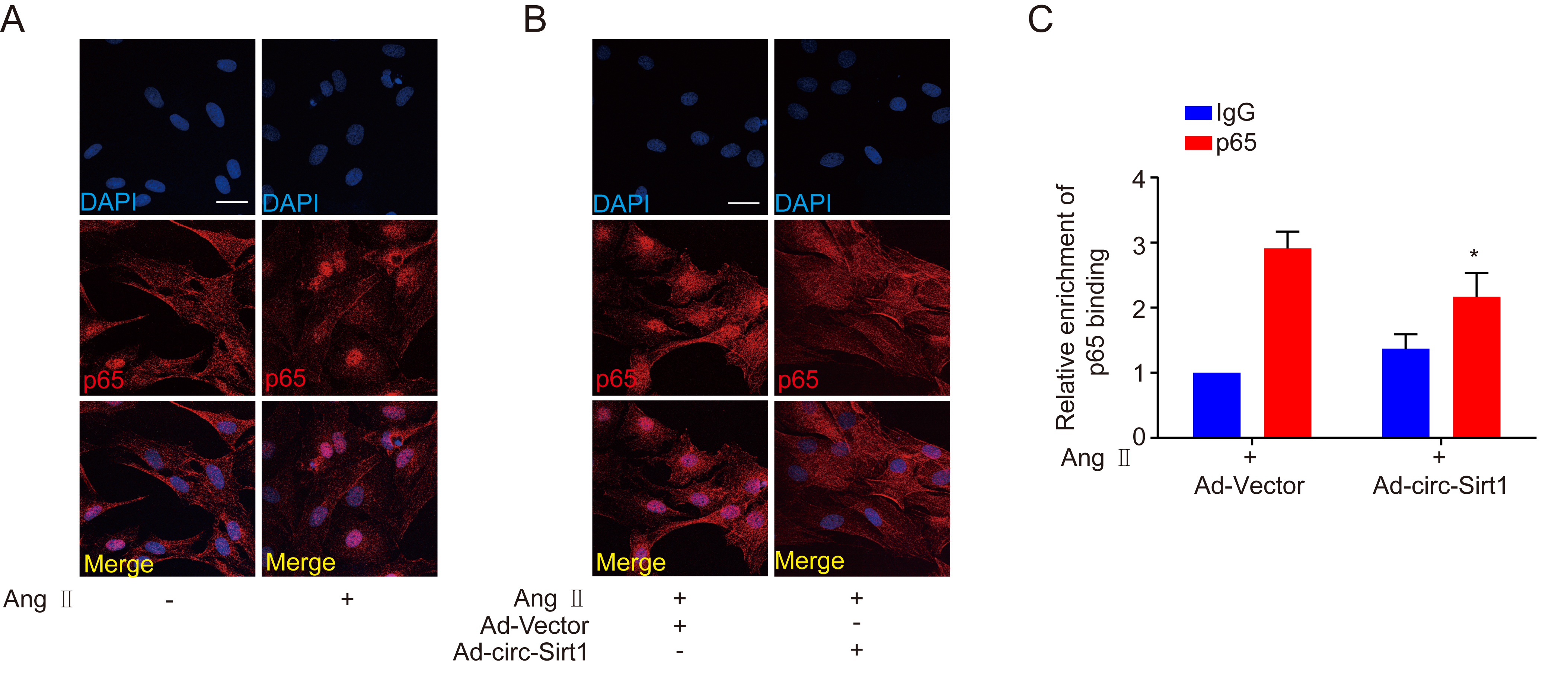


Supplementary Figure 9


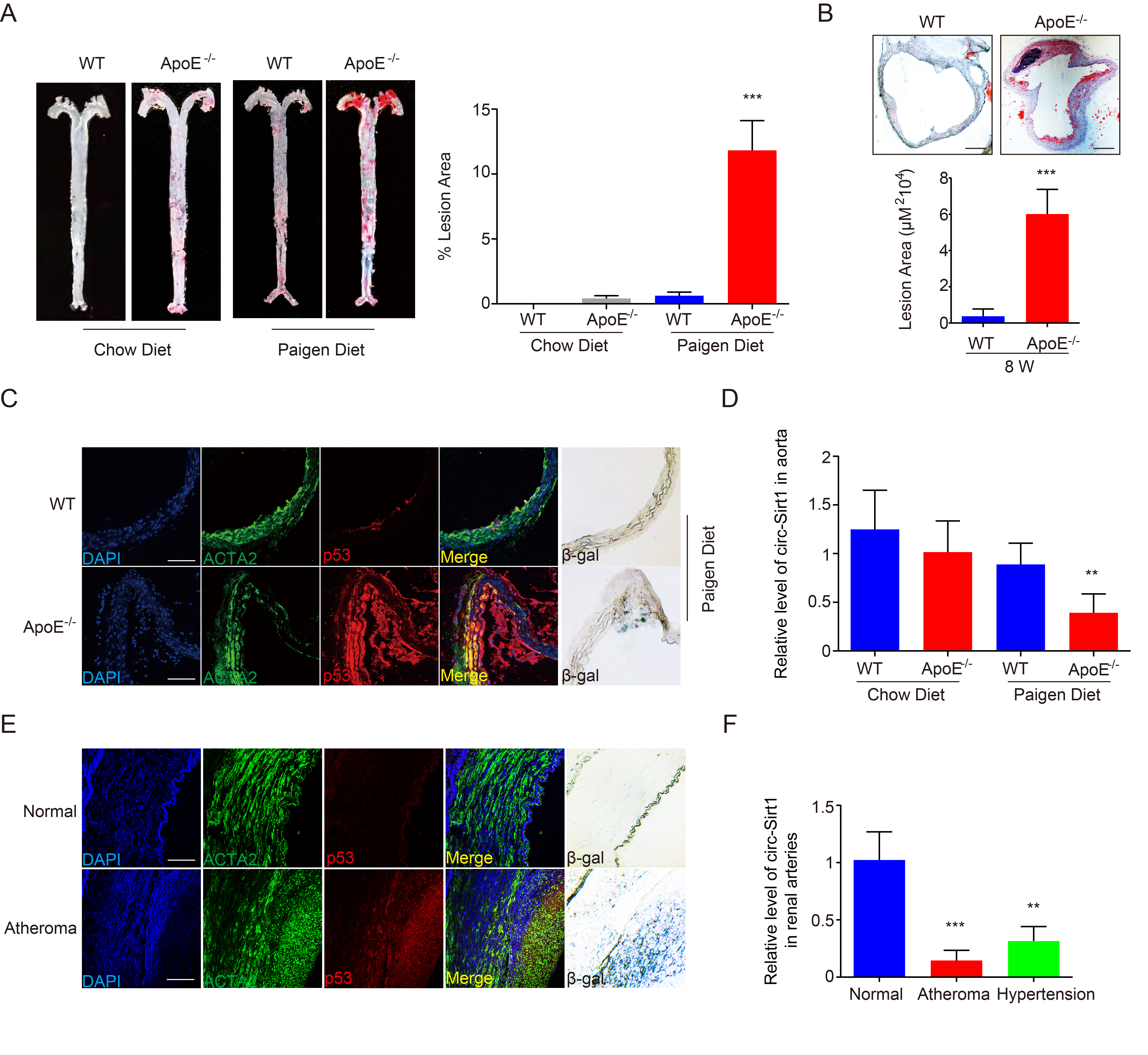


Supplementary Table 1. PCR primer sequence, siRNA sequence, FISH probe sequence and RNA pull down probe sequence

| Primer sequence |  |  |
| --- | --- | --- |
| circ-Sirt1 full length | Forward | CTCTGTGTCACAAATTCATAGCTTTG |
|  | Reverse | ACGGCTGGAACTGTCCGGGATATA |
| circ-Sirt1 | Forward | GTGAAGCTGTTCGTGGAGAC |
|  | Reverse | ATGAAAGCCATTAGTGAGGAGT |
| p53 | Forward | GCAGCACAGGAACCTGGAACTG |
|  | Reverse | AGAAGGGACGGAAGATGACAGAGG |
| GAPDH | Forward | CAACGACCCCTTCATTGACC |
|  | Reverse | ACACCAGTAGACTCCACGACA |
| siRNA sequence |  |  |
| si-Con | Sense | UUCUCCGAACGUGUCACGUTT |
|  | Antisense | ACGUGACACGUUCGGAGAATT |
| si-circ-Sirt1-1 | Sense | UCCAAACAACCUCCUGUUGTT |
|  | Antisense | CAACAGGAGGUUGUUUGGATT |
| si-circ-Sirt1-2 | Sense | CUAAUUCCAAACAACCUCCUTT |
|  | Antisense | AGGAGGUUGUUUGGAAUUAGTT |
| Chip primer |  |  |
| NF-κB binding site | Sense | GCAACATCTCAAGCAGAACCCT |
|  | Antisense | CCCCTAACTGTAGTCGCTACCTA |
| p53 binding site | Sense | CCCCGCCTCCATTTCTT |
|  | Antisense | GTCCCAATCCCAGCAACC |
| Fish probe (biotin) |  |  |
| NC probe |  | UGCUUUGCACGGUAACGCCUGUUUU |
| 18s probe |  | CUGCCUUCCUUGGAUGUGGUAGCCGUUUC |
| circ-Sirt1 probe |  | GGUCAACAGGAGGUUGUUUGGAAUUAGUGCUAC |
| RNA pull down probe (biotin) | |  |
| Oligo | | GTAGCACTAATTCCAAACAACCTCCTGTTGGC |
| circ-Sirt1 | | GCCAACAGGAGGTTGTTTGGAATTAGTGCTAC |

Supplementary Table 2. Proteins identified in whole eluted proteins by MS may specifically interact with circ-Sirt1 compared to Oligo group.

| Protein | oligo Peptide | circ-Sirt1 Peptide | Protein | oligo Peptide | circ-Sirt1 Peptide | Protein | oligo Peptide | circ-Sirt1 Peptide |
| --- | --- | --- | --- | --- | --- | --- | --- | --- |
| PURB | 0 | 8 | HP1BP3 | 2 | 6 | PARP1 | 11 | 18 |
| PURA | 0 | 6 | CCDC59 | 1 | 3 | MYL12B | 5 | 8 |
| TARDBP | 0 | 6 | HADHB | 1 | 3 | RSL1D1 | 8 | 12 |
| MYO1D | 0 | 6 | UBTF | 1 | 3 | H2AJ | 8 | 12 |
| RBM45 | 0 | 5 | DDX18 | 1 | 3 | ARF3 | 2 | 3 |
| POT1 | 0 | 5 | EIF5B | 1 | 3 | WDR12 | 2 | 3 |
| PLCD3 | 0 | 5 | DHX9 | 1 | 3 | CAPZA1 | 2 | 3 |
| HNRNPH3 | 0 | 4 | RPA3 | 3 | 7 | SURF6 | 2 | 3 |
| MSI2 | 0 | 4 | HNRNPAB | 11 | 25 | FAM98A | 2 | 3 |
| DDX25 | 0 | 4 | DDX21 | 4 | 9 | PSMD3 | 2 | 3 |
| TRIP12 | 0 | 3 | HIST2H2AB | 4 | 8 | TLN1 | 2 | 3 |
| PIP5K1A | 0 | 3 | HP1BP3 | 3 | 6 | NOP58 | 7 | 10 |
| MSH2 | 0 | 3 | WDR76 | 3 | 6 | TMOD3 | 5 | 7 |
| COL5A3 | 0 | 3 | CAPZB | 2 | 4 | SGPL1 | 5 | 7 |
| TOP2A | 0 | 3 | GNAI2 | 2 | 4 | TOP1 | 16 | 22 |
| TRP53 | 0 | 2 | HNRNPH1 | 2 | 4 | H2AC20 | 8 | 11 |
| PGAM5 | 0 | 2 | CFL1 | 1 | 2 | HNRNPDL | 8 | 11 |
| RALY | 0 | 2 | NOL12 | 1 | 2 | CKAP4 | 9 | 12 |
| DEK | 0 | 2 | RPS5 | 1 | 2 | TCOF1 | 9 | 12 |
| NGDN | 0 | 2 | TFAM | 1 | 2 | DHX36 | 6 | 8 |
| TERF1 | 0 | 2 | RPL18 | 1 | 2 | RAB1A | 3 | 4 |
| CORO1A | 0 | 2 | SNRPD3 | 1 | 2 | HMGB1 | 3 | 4 |
| RMI1 | 0 | 2 | EIF4A3 | 1 | 2 | GAR1 | 3 | 4 |
| DBN1 | 0 | 2 | CAPZA2 | 1 | 2 | HMGB2 | 3 | 4 |
| UHRF1 | 0 | 2 | RPL15 | 1 | 2 | GTPBP4 | 3 | 4 |
| ELOA | 0 | 2 | TMA16 | 1 | 2 | HNRNPD | 20 | 26 |
| PRPF6 | 0 | 2 | CDC2 | 1 | 2 | RPL36A-PS1 | 7 | 9 |
| TJP1 | 0 | 2 | RNASEH1 | 1 | 2 | TCOF1 | 16 | 20 |
| MYO5A | 0 | 2 | MYL10 | 1 | 2 | RRBP1 | 12 | 15 |
| MYEF2 | 2 | 18 | GPATCH4 | 1 | 2 | H2AZ1 | 8 | 10 |
| DAZAP1 | 1 | 8 | ANXA1 | 1 | 2 | TIAL1 | 8 | 10 |
| PDCD11 | 1 | 6 | PLEKHF2 | 1 | 2 | KRT2 | 8 | 10 |
| NAT10 | 2 | 11 | SLC2A | 1 | 2 | GAPDH | 4 | 5 |
| RPL19 | 1 | 5 | FLNA | 1 | 2 | H1FX | 4 | 5 |
| MSN | 1 | 5 | PRPF8 | 1 | 2 | LRRC59 | 4 | 5 |
| RPL4 | 1 | 5 | MYL12A | 6 | 11 | LIMA1 | 4 | 5 |
| SPTBN1 | 3 | 13 | RPS6 | 6 | 11 | DDX1 | 4 | 5 |
| MRPS15 | 1 | 4 | TIA1 | 5 | 9 | TAF15 | 17 | 21 |
| EIF4A1 | 1 | 4 | NPM1 | 5 | 9 | RPS19 | 9 | 11 |
| SNRNP70 | 1 | 4 | AP2M1 | 5 | 9 | HNRNPA3 | 84 | 102 |
| SSB | 1 | 4 | RPL24 | 4 | 7 | MYH10 | 29 | 35 |
| ABCF1 | 1 | 4 | NOP56 | 7 | 12 | VIM | 20 | 24 |
| LMNA | 1 | 4 | TPM1 | 6 | 10 | HIST1H4A | 15 | 18 |
| COPA | 1 | 4 | PUM3 | 6 | 10 | RBM39 | 10 | 12 |
| ACTN4 | 1 | 4 | IQGAP1 | 6 | 10 | RPL10A | 5 | 6 |
| MYH7 | 1 | 4 | MYO1E | 3 | 5 | RPL6 | 5 | 6 |
| SPTAN1 | 2 | 7 | GSN | 3 | 5 | HNRNPC | 5 | 6 |
| HNRNPM | 28 | 92 | MYO1C | 3 | 5 | RBFOX2 | 5 | 6 |
| CAVIN1 | 3 | 9 |  |  |  |  |  |  |
